# Should brief interventions in primary care address alcohol problems more strongly?

**DOI:** 10.1111/add.12388

**Published:** 2013-11-22

**Authors:** Jim McCambridge, Stephen Rollnick

**Affiliations:** 1 Faculty of Public Health and Policy, London School of Hygiene and Tropical MedicineLondon, UK; 2 Institute of Primary Care and Public Health, School of Medicine, Cardiff UniversityCardiff, UK

**Keywords:** Alcohol, brief intervention, motivational interviewing, primary care

## Abstract

**Background:**

Brief interventions have well-established small effects on alcohol consumption among hazardous and harmful drinkers in primary care, and national large-scale programmes are being implemented in many countries for public health reasons.

**Methods:**

This paper examines data from reviews and draws upon older brief intervention studies and recent developments in the literature on motivational interviewing to consider the capacity of brief interventions to benefit those with problems, including those with severe problems.

**Results:**

Effects on alcohol problems have been shown much less consistently, and evidence cannot be claimed to be strong for any outcomes other than reduced consumption. Combinations of advice and motivational interviewing are a promising target for evaluation in trials, and more detailed studies of the conduct of brief interventions are needed.

**Conclusions:**

We propose that brief interventions in primary care may be more effective if they offer appropriate content in a person-centred manner, addressing patient concerns more directly.

## Introduction

Primary care was the key setting for the major World Health Organization (WHO) collaborative project that developed the Alcohol Use Disorders Identification Test (AUDIT) screening instrument [1] and undertook the first cross-national brief intervention trial [2]. After approximately 30 years of study [3,4], the effects of brief interventions in primary care are, to some extent, now well established [5], while evidence has been much slower to accumulate in other health and non-health settings [6]. Hazardous (i.e. risky drinkers without problems) and harmful drinkers (i.e. those with problems) are consistently helped to drink a little less on average by brief interventions for up to a year, although impacts on alcohol problems are less clear [5]. Brief alcohol interventions have little or no effect on health service utilization [7] and do not appear to impact upon other health-compromising behaviours [8]. The paucity of consistently established wider benefits may partly explain the slow pace of implementation of large-scale programmes based upon the existing evidence base [9,10]. National programmes are now in place across health settings in the United Kingdom, Sweden, Finland and elsewhere, due in part to the need to intervene to reduce alcohol problems at a population level with politically acceptable measures [10]. While this is a considerable achievement in the translation of research into policy and practice, it is also humbling how little is known about key issues [11].

‘Brief intervention’ is a useful umbrella term for two main types of heterogeneous content: brief advice or adaptations of motivational interviewing (MI) [6]. Brief advice appears to be simpler and to lend itself to wide implementation [12], and usually involves trying to persuade the person to drink less [13]. For example, the brief advice evaluated in the recent Screening and Intervention Programme for Sensible drinking (SIPS) trial [14], which has its origins in the advice evaluated in the WHO cross-national trial conducted more than 20 years ago [2], provided information on alcohol and national guidelines, and the benefits of cutting down (or stopping in the case of dependent drinkers) and practical tips. This type of content, focused on the risk behaviour itself, does not enquire about, and thus does not address directly, any problems that someone may be having with their drinking [15]. This approach contrasts sharply with that of MI [16], where asking the person about their situation, and listening carefully to what they have to say, places their concerns or problems at the heart of the conversation. In the face of this contrast, the discussion below will consider the brief intervention evidence base and ask whether we can help those with alcohol problems more effectively.

## Evidence on Problems, Problems with Evidence …

The relative effectiveness of brief advice and adaptations of MI in comparison to each other is largely unknown for any of the three possible target populations of hazardous, harmful and dependent drinkers. We are aware of only one meta-analytic finding suggesting added benefit with MI over advice for alcohol outcomes [17], and further studies are clearly needed. One limitation of many systematic reviews of brief interventions is that they do not evaluate outcomes in relation to intervention content. While some existing reviews identify brief intervention effects on alcohol problems or composite outcome measures, they also suggest diminished effectiveness among those with more severe problems, as effect sizes are lower if dependent drinkers are not excluded [18]. Meta-analytic estimates of effects on alcohol problems assessed with validated instruments are often lacking (e.g. [5]). One review finds that evidence is absent for those who are dependent [19], and a common view is that brief interventions are probably not effective for dependent drinkers [6].

This situation is curious is a number of ways. First, the original studies that pioneered this field, two trials undertaken by Morris Chafetz and colleagues in a Boston emergency room approximately 50 years ago, both found important effects in alcohol-dependent populations [20,21]. Across both studies, approximately 70% of those receiving a referral to out-patient treatment subsequently attended versus approximately 5% who received usual care. Secondly, in the contemporary alcohol treatment literature, motivational enhancement therapy (MET; a multiple session adaptation of MI based on the two-session Drinker's Check-Up, offered to people who are concerned about their drinking [22]), is unsurpassed as a psychosocial treatment, being usually found equivalent in effectiveness to other treatments while also being briefer [23]. It has been established consistently across meta-analytic reviews that MI is no less effective among those with severe problems, including dependence [24].

Brief intervention trials in primary care targeting problem drinkers are very rare. There is no reason why brief psychosocial treatments such as MET, in addition to or as well as pharmacological treatments, should not be delivered in primary care [25,26]. Indeed, it is odd that they have not, as there is obvious unexplored potential. Mike Russell and colleagues' general practitioners (GPs) advice trial [27] was seminal for the alcohol field as well as for the smoking cessation field [6]. While brief advice in primary care is similarly established as modestly effective in both fields [28], the smoking cessation literature is more advanced in the evaluation of brief feedback and counselling interventions for dependent smokers that do not need to be delivered only in specialist services [29,30], and which may be combined effectively with pharmacological treatments [31]. In the alcohol field, the need to rethink the role of specialist services within the health-care system is well recognized [32,33].

It is suggested that there is a prima-facie case that the literature in primary care has been slow to address the needs of problem drinkers. It is also true that existing evidence suggests that harmful drinkers may benefit as much as hazardous drinkers from reductions in consumption following brief interventions, by virtue of the absence of data showing effect modification by severity. It is possible that by including hazardous drinkers who have few or no problems, we inadvertently obscure impacts on problems among those who do have them. It may be necessary to target harmful drinkers directly and/or to design evaluation studies to identify possible impacts on problems only among those who have them.

## Alcohol in Primary Care

Apart from in emergency contexts, hazardous or harmful drinking is rarely the primary reason for attendance in primary care, and sensitive engagement on this subject is necessary if people are to become willing to discuss it in depth. Box 1 provides two brief pen-portraits of possible presentations. We suggest that Jill and John may be best helped by supporting their exploration of their situation, rather than having an initial or sole focus on trying to get them to drink less. Brief advice is simply not designed for careful engagement in discussion, although it may stimulate some useful thinking. A more sophisticated approach to advice-giving may be called for that is not very distinct from counselling. More than two decades ago, this was the direction pointed to by the FRAMES (Feedback, Responsibility, Advice, Menu, Empathy, Self-efficacy) acronym [34–36], although this did not lead successfully to process studies as was originally hoped, and as a result we still know little about the content of effective alcohol advice [37]. We cannot go on like this.

**Box 1: How should we talk to Jill and John about alcohol?**

Jill is a 20-year-old student who goes out with friends to pubs and clubs most weekends. She enjoys drinking heavily or getting drunk most Friday and Saturday evenings, as do most people she knows. She's done a few things she'd prefer to forget, and really doesn't like her hangovers, which sometimes completely wipe out the next day. She is well aware of the recommended limits and is happy to talk about her drinking because it's no big deal. She visits the practice to get her contraceptive prescription renewed.

John is a middle-aged man who sometimes gets mild panic attacks when around traffic. As well as sharing a bottle of wine with his wife over dinner a couple of times a week, he drinks in the evening and at weekends. He also sometimes drinks to calm his nerves to make the bus journey home from work easier, and when he does so, ends up drinking for the rest of the evening. He doesn't know what he thinks about his drinking and would probably prefer to avoid discussing it. He visits the practice to have his prescription renewed.

More is known about what helps in MI where process studies have been developed successfully based on validated instruments, which reveal the complexities of these discussions [38–41]. We are at an early stage in understanding effective content [42], and the need to develop understanding of mechanisms of behaviour change extends far beyond both MI and alcohol. What might appear to be little things, such as conveying willingness to help, reflecting back what you have heard, being curious to know more, paying very careful attention to language, asking permission to offer views, the timing of key utterances and following-up on responses, all appear to be helpful [16]. Together, these are characteristic but far from unique elements of the style of MI, and they also provide guidance for ways in which advice may be given when influenced by this approach. This would also avoid assuming that decisions to change have been made or that goals for behaviour change, whether abstinence for dependent drinkers or nationally recommended risk thresholds for non-dependent drinkers, are the right ones for every person. It is assumed that this approach takes longer to implement than advice. There have been different models of brief MI for more than 20 years (e.g. [22,43,44]), and there has also been concern that it is too complex for practitioners to learn and deliver well. MI itself has become simpler, identifying four processes of engagement, focusing, evoking and planning [16] in ways that provide content for a new generation of models of how to conduct MI when time is short, as is often the case in primary care. Even where time is short there is scope for, and potential benefit in, being person-centred.

## Future Research

It is suggested here that brief interventions could encourage people with alcohol problems to tell us what their problems are, so that help can be provided to think these through in so doing, in order to help initiate or better support efforts at change. While this proposal is congruent with the highly person-centred nature of MI and lends itself to evaluation in trials, there are also other ways in which this type of intervention content can be delivered and evaluated, and there is a particular need for detailed study of how discussions about alcohol in primary care actually take place. This means using validated instruments such as Motivational Interviewing Treatment Integrity (MITI) [45]. Where adaptations of MI are only loosely based on this well-specified approach, or where MI fidelity is not high, we would expect any effects to be indistinguishable from advice [46].

It is also important to acknowledge how limited is the science of helping practitioners prepare for behaviour change discussions, which are unavoidably complex whichever intervention approach is being used. One hour of training was provided to deliver advice in the SIPS primary care trial, which may be insufficient to be effective [47]. The Phase III REsearch Evaluating Migraine Prophylaxis Therapy 1 (PREEMPT) trial of training primary care practitioners also found no effects on alcohol outcomes, and it remains unclear which skills practitioners really need to possess, and how they may best acquire this learning [48]. There is obvious merit in eliciting the experiences and the views of practitioners themselves [49]. Apparently simple interventions may be deceptively alluring on cost and implementation grounds, although they will not be worth implementing if they are not effective [42]. Quick fixes will sometimes be in accord with the needs of the client, but often may not be. Arguably, they are optimally designed when the complexities inherent in these discussions [50] are better understood than they are now.

Alcohol problems do not occur in isolation from other difficulties. Many people drink excessively, smoke and have other life-style difficulties. There is little current evidence on where and how multiple problems can be addressed by brief interventions [51]. On-line trials indicate that large numbers of people are prepared to seek help with alcohol [52], and it remains to be established what role primary care has in delivering these interventions in the era of the internet. As screening and simple feedback can be delivered widely on-line, we suggest here that primary care may provide the key setting in which widely available face-to-face discussions can be offered. One final research question well worth answering is how demand for brief interventions may be nurtured [53], so that those who may wish to consider their drinking by discussing it will actually seek them out. We should ask them.
